# Are Treatments More Effective than Placebos? A Systematic Review and Meta-Analysis

**DOI:** 10.1371/journal.pone.0062599

**Published:** 2013-05-15

**Authors:** Jeremy Howick, Claire Friedemann, Maria Tsakok, Robert Watson, Teresa Tsakok, Jennifer Thomas, Rafael Perera, Susannah Fleming, Carl Heneghan

**Affiliations:** Department of Primary Care Health Sciences, University of Oxford, Oxford, United Kingdom; University of Louisville, United States of America

## Abstract

**Background:**

Placebos are widely used in clinical practice in spite of ethical restrictions. Whether such use is justified depends in part on the relative benefit of placebos compared to ‘active’ treatments. A direct test for differences between placebo and ‘active’ treatment effects has not been conducted.

**Objectives:**

We aimed to test for differences between treatment and placebo effects within similar trial populations.

**Data Sources:**

A Cochrane Review compared placebos with no treatment in three-armed trials (no treatment, placebo, and treatment). We added an analysis of treatment and placebo differences within the same trials.

**Synthesis Methods:**

For continuous outcomes we compared mean differences between placebo and no treatment with mean differences between treatment and placebo. For binary outcomes we compared the risk ratio for treatment benefit (versus placebo) with the risk ratio for placebo benefit (versus no treatment). We conducted several preplanned subgroup analyses: objective versus subjective outcomes, conditions tested in three or more trials, and trials with varying degrees of bias.

**Results:**

In trials with continuous outcomes (*n* = 115) we found no difference between treatment and placebo effects (MD = −0.29, 95% CI −0.62 to 0.05, *P* = 0.10). In trials with binary outcomes (*n* = 37) treatments were significantly more effective than placebos (RRR = 0.72, 95%CI = 0.61 to 0.86, *P* = 0.0003). Treatment and placebo effects were not different in 22 out of 28 predefined subgroup analyses. Of the six subgroups with differences treatments were more effective than placebos in five. However when all criteria for reducing bias were ruled out (continuous outcomes) placebos were more effective than treatments (MD = 1.59, 95% CI = 0.40 to 2.77, *P* = 0.009).

**Conclusions and Implications:**

Placebos and treatments often have similar effect sizes. Placebos with comparatively powerful effects can benefit patients either alone or as part of a therapeutic regime, and trials involving such placebos must be adequately blinded.

## Introduction

To what standard must a placebo be held, if not that it equals the active treatment? [1].

A recent Cochrane Review allegedly “did not find that placebo interventions have important clinical effects” [2]. If placebos have negligible effects then their widespread use in clinical practice seems unjustified [3]–[6]. Indeed this is just what the authors conclude. They “suggest that placebo interventions are not used outside clinical trials” [2].

**Figure 1:**
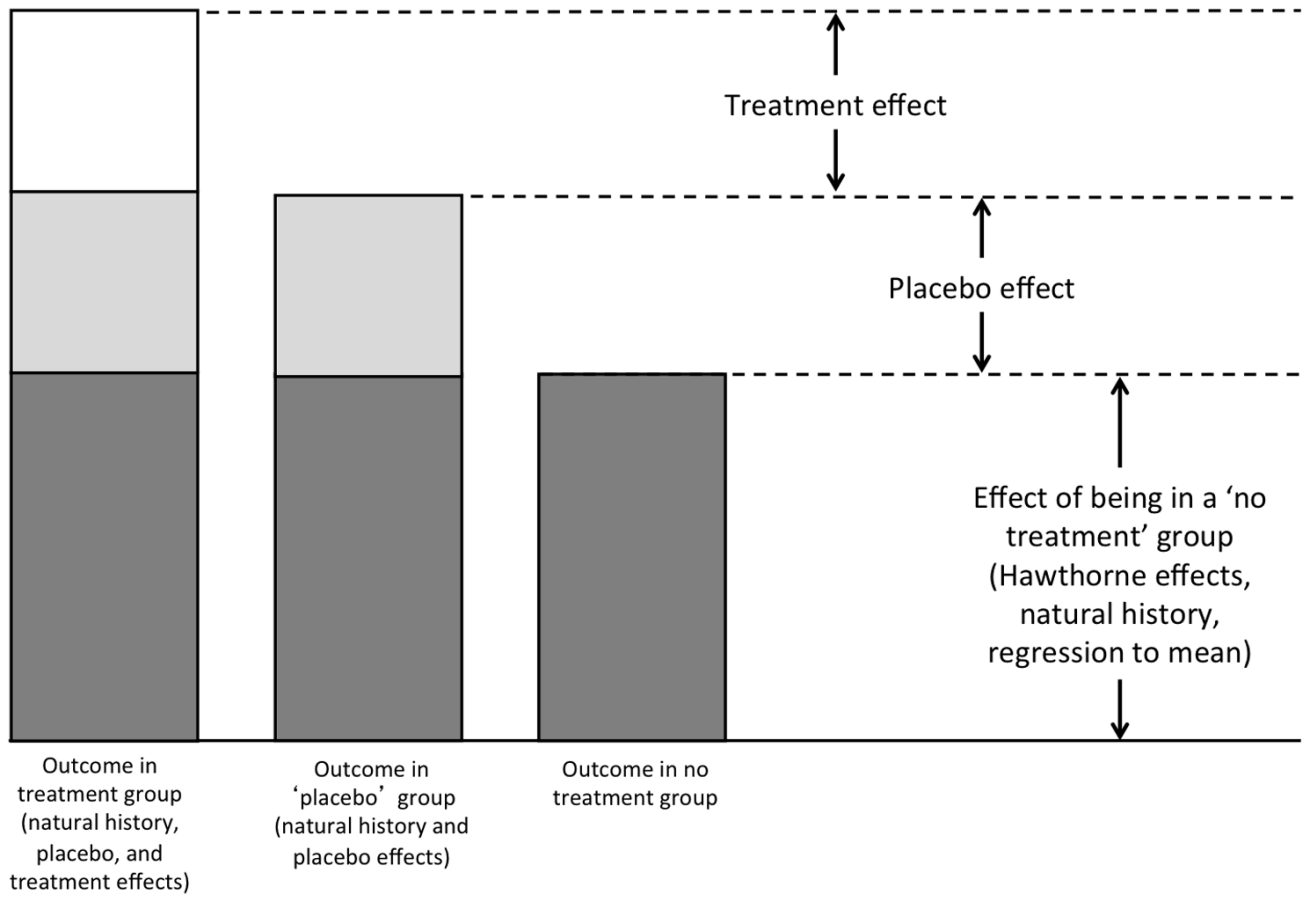
Outcomes in treatment, placebo, and no treatment groups.

By contrast with the Cochrane Review. earlier studies noted a third of patients recovered after taking placebos and inferred that placebo effects caused the cure [7], [8]. However improvement after taking the placebo could have been due to natural history – many illnesses fluctuate or go away without treatment [9]. Hence accurate measurements of placebo effects must involve comparison with untreated groups (see Figure 1). This is just what the authors of the Cochrane Review did and they therefore exposed early claims about placebo effects as exaggerated.

**Figure 2:**
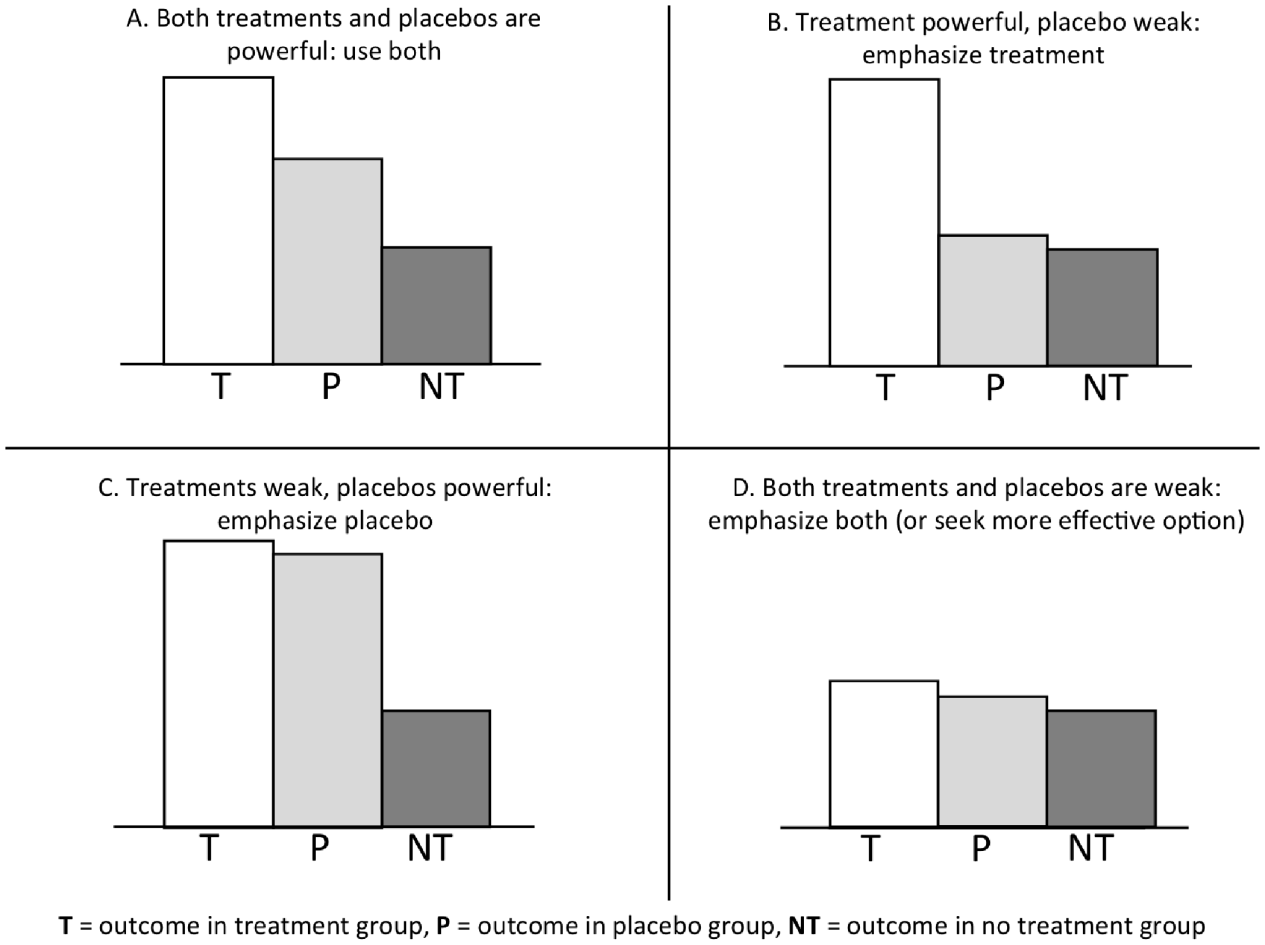
The importance of knowing the relative benefits of treatment and placebo effects.

Several potential methodological problems with the Cochrane Review have already been discussed [10]–[18]. A problem that has hitherto been ignored is that the results from the Review alone do not warrant claims about the usefulness of placebos in clinical practice. Just as clinical usefulness of treatments depends on how they compare with other interventions for the same condition, so the clinical usefulness of placebos requires comparison with treatments (see Figure 2) [19], [20]. Even modestly effective placebos may benefit patients if their effects are at least as large as treatment effects. Likewise, even very effective placebos may not be worthwhile exploiting if treatment effects are much greater. In any case the rationale for using placebos (or not) depends in part on the relative benefit of placebos compared with treatment.

In this systematic review we aimed to test for statistically significant differences between placebo and treatment effects within the same randomized trials.

## Methods

### Eligibility Criteria, Information Sources, Search, Study Selection, and Risk of Bias in Individual Studies

The least biased method for measuring comparative effectiveness of different treatments is within the same trials [20]–[22]. A Cochrane Review measured placebo effects within three-armed trials (no treatment, placebo, and treatment) [2]. The Review did not include any data about outcomes in ‘active’ treatment groups. Taking the same trials, we extracted data about treatment effects and added a comparison of treatments and placebos. The review excluded non-randomized trials, trials with unblinded outcome assessment, and trials reporting >50% dropout rates. We accepted these criteria as they reduce the risk of serious bias [23]–[26].

### Defining the Placebo

A barrier to estimating placebo effects is that ambiguity surrounds the ‘placebo’ concept [27]–[32]. Placebos are often characterized as inactive or nonspecific when in fact they can be active and have specific effects, especially for relieving pain [33], [34]. A recent attempt to clarify the placebo concept involves classifying placebos as either ‘pure’ or ‘impure’ [4], [5], [35]–[40]. Pure placebos are interventions such as sugar pills (which are available commercially [41]) or saline injections allegedly without direct pharmacologically active ingredients. Impure placebos are interventions with clear efficacy for certain conditions but are prescribed where their efficacy is unknown. Examples include antibiotics for suspected viral infections [4], off-label prescriptions, and some complementary treatments lacking a sound evidence-base [42], [43]. However, the pure/impure dichotomy is a rough guide at best. Just as antibiotic treatments can function as treatments for bacterial infections or placebos for viral infections, so sugar is not inert with respect to diabetes [28], and saline solution has many clinical uses [44]. Indeed few substances (if any) are completely inactive for all conditions [45]. The problems with characterizing placebos has led some to conclude that there is no logic in the placebo concept [27], or even that term ‘placebo’ should be abandoned [29]. Yet without an adequate definition it seems difficult to measure placebo effects accurately because we won’t know what we are measuring.

Fortunately in the context of a placebo controlled trial the conceptual problems are somewhat constrained. Placebo controls are usually treatments that appear similar to the experimental treatments, but that lack their characteristic components [46]. Following the 2010 Cochrane Review of placebo effects, we adopt a pragmatic approach and refer to placebos as interventions described as such in the context of a randomized trial [2]. To be sure this does not entirely solve the problem. For example, olive oil was used in ‘placebo’ capsules for trials of cholesterol-lowering agents before there was evidence that olive oil reduced cholesterol [45]. However the problem of inadequate or illegitimate placebos in clinical trials may be rare [33]. Moreover the pragmatic approach has two important advantages. First, it avoids the requirement to justify the tenuous distinction between pure and impure placebos. Second it is more useful: patients, doctors, and policy makers care more about whether particular interventions are effective and ethical than whether these treatments carry the label ‘placebo’. Practical implications of our results must therefore involve adequate descriptions of placebo (and treatment) interventions [33], [47].

### Data Collection Process and Data Items

We obtained full text copies of articles and extracted data to an Excel template that was piloted by two authors (JH, CH). Four reviewers (MT, TT, JW, RW) extracted authors’ names, addresses, publication year, placebo type, outcome type, and outcomes in all three groups. Two authors (JH, CF) did the second extraction. We contacted authors of included studies when reported outcome data were inadequate for meta-analysis. Discrepancies were resolved by discussion.

### Summary Measures and Synthesis of Results

For continuous outcomes the treatment effect was defined as the standardized mean difference between an unwanted outcome in the treatment group and an unwanted outcome in the placebo group (T–P). The placebo effect was defined as the standardized mean difference between an unwanted outcome in the placebo group and an unwanted outcome in the no treatment group (P–NT) [2]. To test for a difference between treatment and placebo effects we took the null hypothesis to be that there was no difference between treatment (T–P) and placebo (P–NT) effects (see Appendix 1). A negative value of the test statistic was taken to indicate the treatment effect was greater than the placebo effect. We used RevMan (version 5) to calculate the two-tailed *P*-value and 95% confidence intervals.

For binary outcomes we measured the treatment effect by dividing the risk ratio in the treatment group by the risk ratio in the placebo group. The placebo effect was measured by dividing the risk ratio in the placebo group by the risk ratio in the no treatment group [2]. Using a method justified elsewhere [48], we took treatment and placebo effects to differ when the ratio of risk ratios (RRR) deviated from unity (see Appendix 2). Values greater than one indicated treatment effects were greater than placebo effects.

Following the methods used in the Cochrane Review of placebo effects, for crossover trials we only used data from the first period. Where this was impossible we used the summary data as though they had been derived from a parallel trial. For placebo effects we chose final values where possible, or change from baseline if these were the only available data. When there was more than one ‘active’ treatment group, we chose the primary intervention as defined by authors in the paper. Where a primary outcome was unclear we combined data from both treatment groups. We expected heterogeneity and calculated the pooled results with a random effects model. We estimated heterogeneity using the I-squared test.

We replicated several key preplanned subgroup analyses that were also done as part of the Cochrane Review of placebo effects. We divided both continuous and binary outcomes into trials involving subjective (patient-reported) and objective (observer-reported) measures, we examined whether conditions tested in three or more trials, and we tested whether trials with different degrees of methodological quality (allocation concealment, dropout rate exceeding 15%, sample size less than 50, and a combination of all these) could be distinguished. These subgroups were all chosen at the protocol stage so there was no need for a correction. To reduce the chances of spurious correlations we required *P*-values lower than 0.01 to announce statistical significance.

## Results

### Study Selection, Study Characteristics, and Risk of Bias within Studies

We analysed 152 published reports with sufficient data to calculate effect sizes (37 with binary, 115 with continuous outcomes) involving 11,747 participants (placebo versus no treatment comparison) and 12,576 participants (treatment versus placebo comparison). Appendix 3 contains a list of all included studies. The study characteristics and risk of bias have been reported previously [2].

### Continuous Outcomes

**Figure 3:**
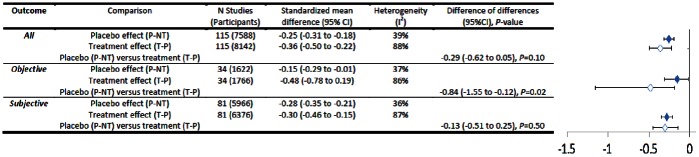
Placebo versus treatment effects (continuous outcomes).

**Figure 4:**
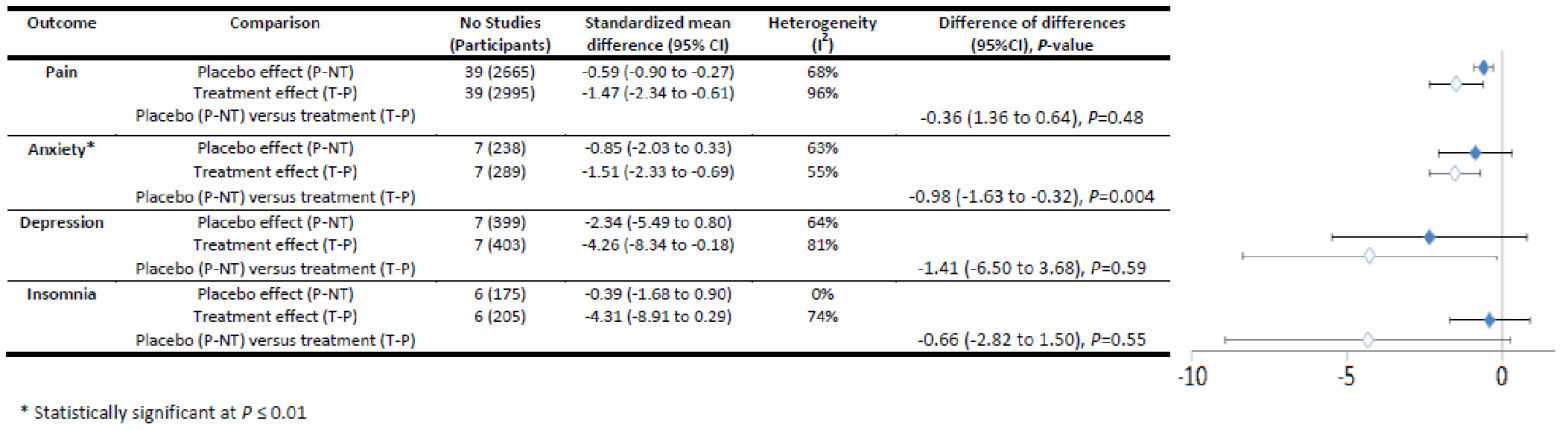
Conditions tested in three or more trials (continuous outcomes).

**Figure 5:**
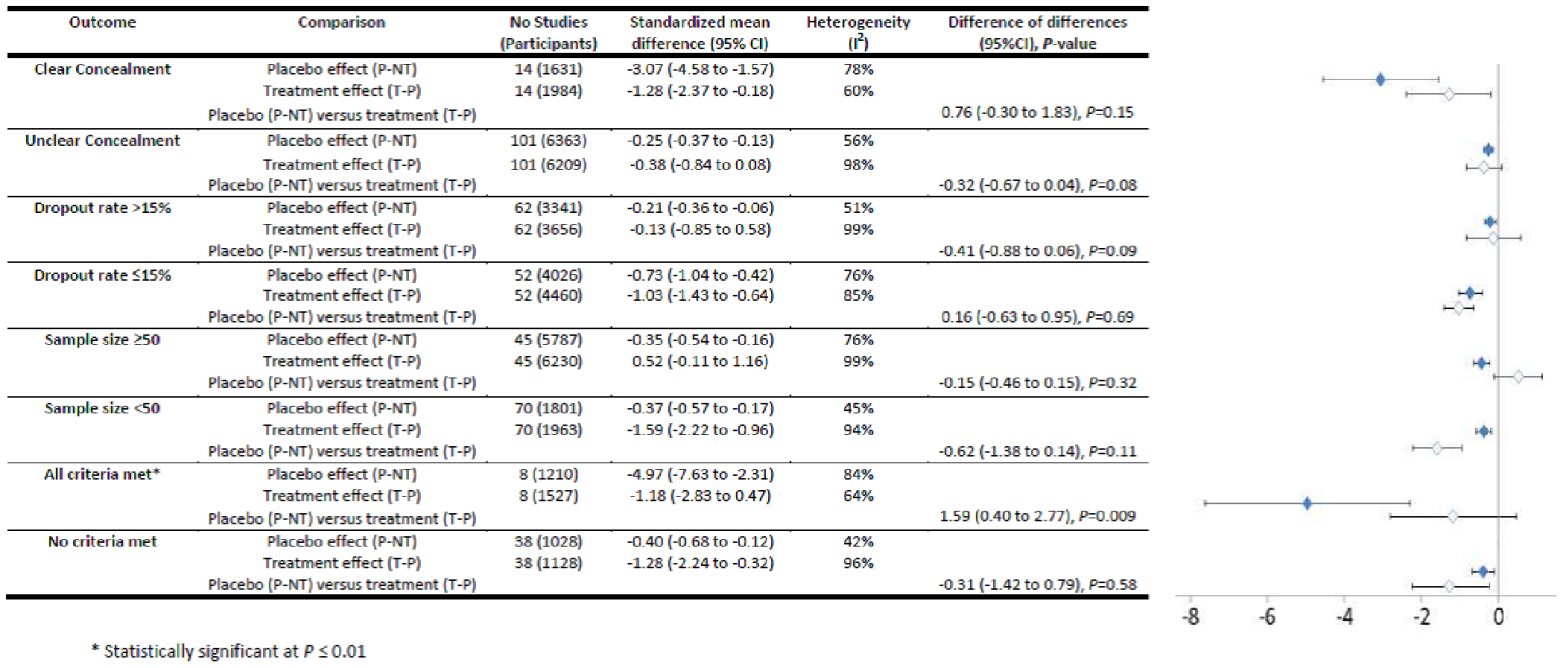
Trials with varying degrees of bias (continuous outcomes).

We found no statistically significant difference between placebo and treatment effect sizes in all trials with continuous outcomes (MD = −0.29, 95% CI = −0.62 to 0.05, *P* = 0.10) (see Figure 3). This held true for all but two out of 14 subgroup analyses. Treatments had borderline statistically significant advantages compared with placebos for objective outcomes (*n* = 34, MD = −0.84, 95% CI = −1.55 to −0.12, *P* = 0.02) but there was no difference in trials with subjective outcomes (*n* = 81, MD = −0.13, 95% CI = −0.51 to 0.25, *P* = 0.50). Four conditions were tested in at least three trials: pain (*n* = 40), depression (*n* = 7), insomnia (*n* = 6), and anxiety (*n* = 7) (see Figure 4). There was no difference between treatment and placebo effect sizes in any of these apart from anxiety, where treatment effects were greater (MD = −0.98, 95%CI = −1.63 to −0.32, *P* = 0.004). In trials with varying degrees of bias treatment and placebo effects were usually similar (see Figure 5). However in trials where all criteria for ruling out bias were met (*n* = 8) placebos were more effective than treatments (MD = 1.59, 95% CI = 0.40 to 2.77, *P* = 0.009).

### Binary Outcomes

**Figure 6:**
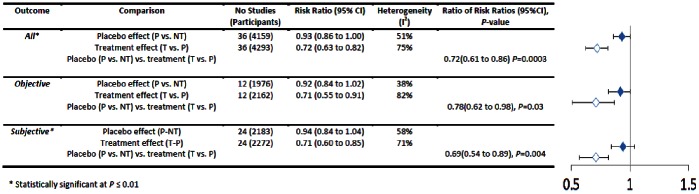
Placebo versus treatment effects (binary outcomes).

**Figure 7:**
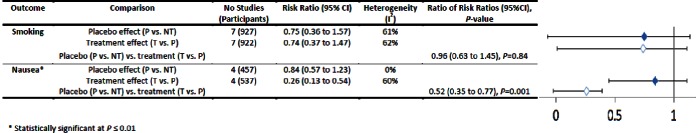
Conditions tested in three or more trials (binary outcomes).

**Figure 8:**
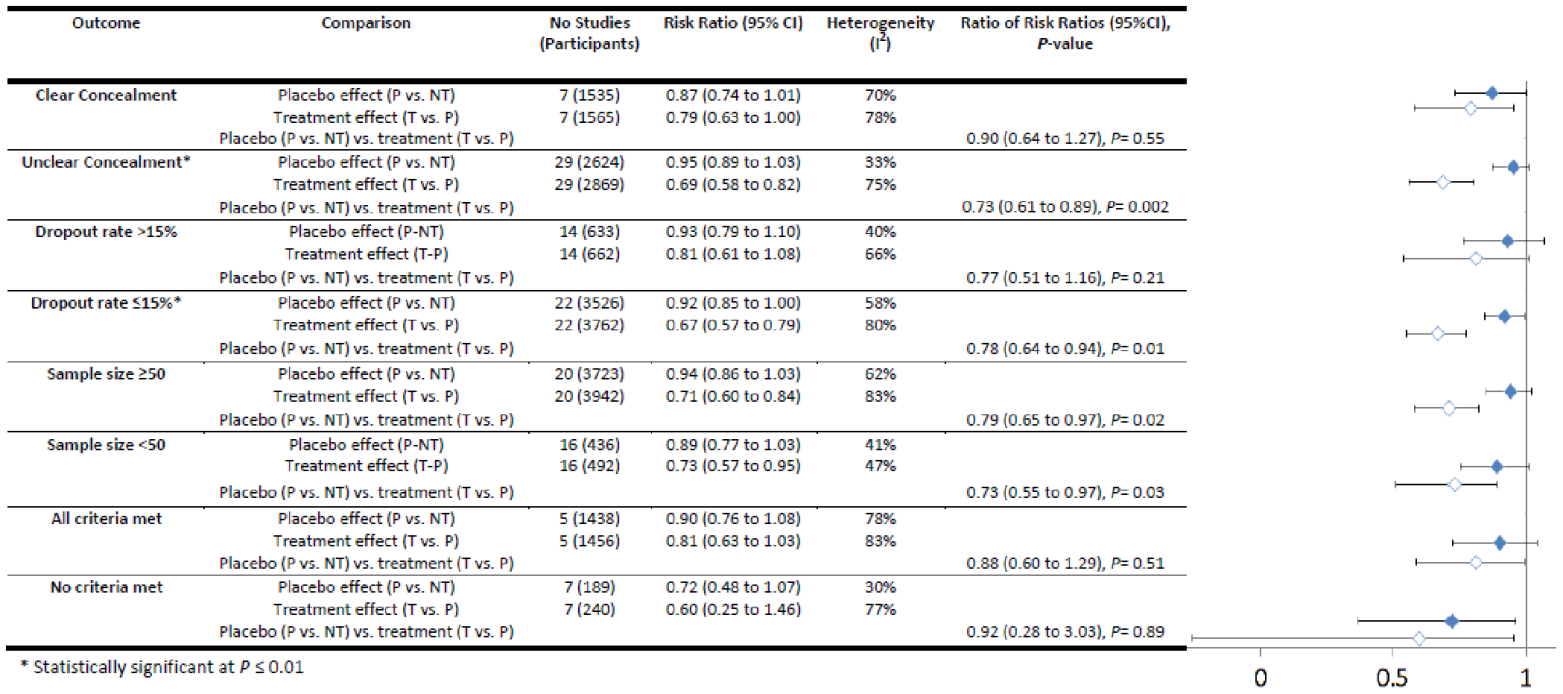
Trials with varying degrees of bias (binary outcomes).

In all trials with binary outcomes treatment effects were greater than placebo effects (RRR = 0.72, 95%CI = 0.61 to 0.86, *P* = 0.0003) (see Figure 6). However placebo and treatment effects were not different in 8 out of 12 subgroups. Treatments had greater benefits than placebos in trials with subjective (*n* = 25, RRR = 0.69, 95%CI = 0.54 to 0.89, *P* = 0.004) but only borderline statistical significance in trials with objective outcomes (*n* = 12, RRR = 0.78, 95%CI = 0.62 to 0.98, *P* = 0.03, respectively). Two conditions were tested in at least three trials: smoking (*n* = 7) and nausea (*n* = 4). Treatments were more effective than placebos for treating nausea (RRR = 0.52, 95%CI = 0.35 to 0.77, *P* = 0.001) but not smoking (RRR = 0.96, 95%CI = 0.63 to 1.45, *P* = 0.84) (see Figure 7). Treatments were more effective than placebos in trials with unclear allocation concealment (*n* = 29, RRR = 0.73, 95%CI = 0.61 to 0.89, *P = *0.002), and where dropout rates were less than or equal to 15% (*n* = 22, RRR = 0.78, 95%CI 0.64 to 0.94, *P* = 0.001), but not in subgroups with other degrees of bias (see Figure 8).

## Discussion

### Summary of Evidence and Comparison with Relevant Literature

We found placebos often had as great a benefit over no treatment as treatments had over placebos. In trials with binary outcomes treatment effects were usually greater than placebo effects, and in trials with continuous outcomes and a low risk of bias placebo effects were greater than treatment effects. Our results are consistent with other reviews suggesting placebos are greatest in trials with continuous outcomes [49]. By providing a direct comparison of placebo and treatment effects, clinicians and policy makers are better able to make decisions about where to allocate scarce resources.

The favourable comparison of placebos with treatments in continuous but not binary outcomes might be explained by three factors: (1) bias, (2) types of ailment tested in trials with continuous outcomes, and (3) problems with dichotomizing outcomes.

#### Bias and exaggeration of treatment and placebo benefits

All trials suffer from some bias. In the context of our main hypothesis the interesting concern about bias is whether confounding is more likely to exaggerate treatment or placebo effects. Both theoretical considerations and our data suggest that more powerful biases tend to overstate treatment effects. The main bias tending to exaggerate placebo effects is response bias. Recall that placebo versus no treatment comparisons cannot be blinded: untreated patients know they are not being treated. Polite patients taking the placebo could report improvement to please investigators although no benefit was actually felt [2], [50], [51]. Similar problems might arise because caregivers and observers are unblinded [46]. These may artificially inflate apparent placebo benefits. Other forces, however, will lead to underestimating placebo effects. For example the ‘untreated’ groups in our analysis involved contact with therapists, maintenance of therapy, and other forms of standard care [11]. Hence some ‘untreated’ groups could have experienced (Hawthorne and context) effects [30], [52]–[54], leading to underestimating placebo power. Indeed a recent systematic review found that untreated groups experienced a 24% improvement compared with baseline [55], which is unlikely to be wholly due to natural history or regression to the mean.

Other biases affect reported treatment benefits. While many treatment versus placebo comparisons are described as blinded, evidence suggests that blinding is rarely successful [56]–[60]. If a trial is unsuccessfully blinded, patients who know they are in the placebo group may drop out, or fail to report recovery. Patients with ailments such as pain or depression could develop negative feelings about having been given a ‘mere’ placebo and actually experience a worsening of their symptoms. Meanwhile patients who know they are receiving the experimental treatment may exaggerate reports of benefits or even (in the case of pain or depression) actually experience improvements [46], [59]. Finally, negative results (for treatment benefit) are less likely to be published [61]. Powerful placebo effects are one cause of negative results so trials with large placebo effects might be less likely to be published. If more biases tend to exaggerate treatment effects [24], [62], we would expect placebo effects to be relatively stronger than treatments in trials with a low risk of bias. This is precisely what we found for continuous outcomes. Future research into trials with a low risk of bias is warranted to confirm our findings.

#### Conditions that are placebo responsive are more likely to use subjective outcomes

Another likely reason why the relative benefit of placebos was greater in trials with continuous outcomes is that the ailments we know to be placebo responsive such as pain and depression are usually measured on continuous scales [54], [63], [64]. Hence the greater placebo effects in these trials could be due to the disorder rather than the outcome type [2], [49].

#### Dichotomizing outcomes leads to underestimating effects

The third potential explanation for the discrepancy between results in continuous and binary outcomes is that dichotomizing outcomes reduces power [16], [65], [66]. If placebos reduce pain by 20% on a 10–point scale, and we dichotomize to require a reduction of 25% to count as an event, then we obscure effects inferior to 25%. This will reduce the power of trials with binary outcomes, and hence the power of meta-analyses involving such trials to detect effects. Examining the evolution of the Cochrane Review of placebo effects as it was updated to include more trials lends credibility to this interpretation. The first (2001) version of the review included 32 trials with binary outcomes and the relative risk was not statistically significant (0.95, 95%CI 0.88 to 1.02). When the review was updated in 2010 to include 44 trials with binary outcomes, the placebo effect reached statistical significance (0.93, 95%CI 0.88 to 0.99). (Aside: in spite of placebo effects reaching statistical significance in the updated review, the authors failed to modify their sceptical conclusions regarding the strength of placebo effects.).

### Strengths and Limitations of this Review

We did not get access to 7 studies (binary outcomes) and 43 studies (continuous outcomes) included in the Cochrane Review. This was expected given the Cochrane Review began almost 15 years ago and some data or authors were no longer accessible. In terms of direction of effect, size of effect, and statistical significance our placebo effect estimates were the same as those in the Cochrane Review for all but one of the 28 comparisons. In the single comparison where our results differed, we did not find a statistically significant difference between placebo and no treatment in all trials with binary outcomes (RR 0.93, 95%CI 0.86 to 1.00) whereas the Cochrane review did (RR 0.93, 95%CI 0.88 to 0.99). Because our point estimate was the same the difference was likely to be related to power.

There are also three issues to consider when generalizing our results to clinical practice. First, interventions tested in clinical trials may be unrepresentative of treatments used in routine practice. In routine practice many interventions are known to be effective and therefore untested in trials [46], [67]. Hence the trials in our review may be skewed by treatments that are, on average, less effective than treatments use in routine practice. However a related phenomenon about placebo effects in the context of blinded trials may balance out this concern. In routine practice a doctor (hopefully) believes the treatments they provide are effective and patients share these positive beliefs. These positive beliefs can exaggerate placebo effects [53]. By contrast in a double blind trial neither patients nor caregivers know whether the intervention is a placebo or a ‘real’ treatment. Hence a component of the placebo (positive belief effects) may be reduced in the trials included in our review [68], [69]. Second, our study was about intervention effects within clinical trials, and effects could differ between trials and practice. However it seems impossible to study placebo effects in clinical practice without introducing an experimental setting. Hence the best we can do is infer findings about placebo effects from trials. Third, placebo treatments in clinical practice are often considered unethical because they allegedly require deception (telling the patient it is a ‘real’ treatment) [3], [70]. By comparison, trial patients give their informed consent. Therefore any extrapolation from our study to routine practice must be done ethically.

A final limitation is that the heterogeneity of treatments, placebos, and ‘no treatments’ used in the review may call into question the justification for pooling results. For example the placebo treatments in our studies included placebo injections, placebo acupuncture, and placebo pills (among many others). These different treatments have been shown to have different effects. Sham injections and acupuncture are more effective than placebo pills [71], [72], and within placebo pills, the colour [73], and perceived cost can influence the effect [74]. Placebo interventions can even produce negative effects in which case they are referred to as ‘nocebos’ [70], [75]. Certainly any practical ramifications of this study must be targeted towards particular conditions and involve adequate descriptions of active [29], [46], [47], and placebo interventions [33], [34]. Our subgroup analyses provides preliminary information about relative placebo and treatment effects for treating specific conditions, and further research into which placebos are most beneficial for various conditions is warranted.

### Implications for Clinical Trials and Practice

The clinical usefulness of placebos requires comparison with treatments and we found that placebo effects are often similar to treatment effects. Trials involving such placebos must be adequately blinded [59], [76], and dichotomizing outcomes in trials with weaker interventions will lead to a loss of power to detect effects. Because the placebo effect is part of the overall treatment effect our findings do not imply that placebos – even powerful placebos – should replace treatments. Rather, this study shows that patients will benefit if doctors exploit relatively powerful placebos either alone or as part of a therapeutic regime. A clear case where placebos might be used for clinical benefit is pain, where placebo effects are similar in magnitude to treatment effects. Meanwhile current ‘active’ treatments for pain such as non-steroidal anti-inflammatory drugs (NSAIDs) and occasional opiates [77] have questionable efficacy in the long term and common and well described adverse effects [78], [79]. On the other hand small relative placebo benefits may be not be merit allocation of scarce healthcare resources. Rational decisions about allocating resources to placebo interventions depends on the direct comparison of placebos and treatments provided in this study.

## Supporting Information

Appendix S1 Hypothesis test for continuous outcomes.
(DOCX)

Appendix S2 Hypothesis test for binary outcomes.
(DOCX)

Appendix S3 References to studies included in this review.
(DOCX)

Flowchart S1 PRISMA flowchart.
(DOC)
