# Correction: Are Treatments More Effective than Placebos? A Systematic Review and Meta-Analysis

**DOI:** 10.1371/journal.pone.0147354

**Published:** 2016-01-15

**Authors:** Jeremy Howick, Claire Friedemann, Maria Tsakok, Robert Watson, Teresa Tsakok, Jennifer Thomas, Rafael Perera, Susannah Fleming, Carl Heneghan

Among studies with continuous outcomes in our systematic review comparing placebo effect sizes with treatment effect sizes we calculated mean differences between to estimate placebo effects, treatment effects, and the difference between placebo and treatment effects. In fact the standardized mean difference should have been used. There was no error in the calculations among studies with binary outcomes. We have corrected this error and revised Figs 3, 4 and 5 from our review here. Correcting the error strengthened our conclusion that there is rarely a statistically significant difference (at *P* = 0.05 or lower) between the magnitude of placebo effects and the magnitude of treatment effects. In the original review placebo effect sizes and treatment effect sizes did not differ by a statistically significant amount in the three subgroup analyses: all studies with objective outcomes (treatment effects larger), studies of anxiety treatments (treatment effects larger), and studies where all criteria for ruling out bias were met (placebo effects larger).

**Fig 3:**
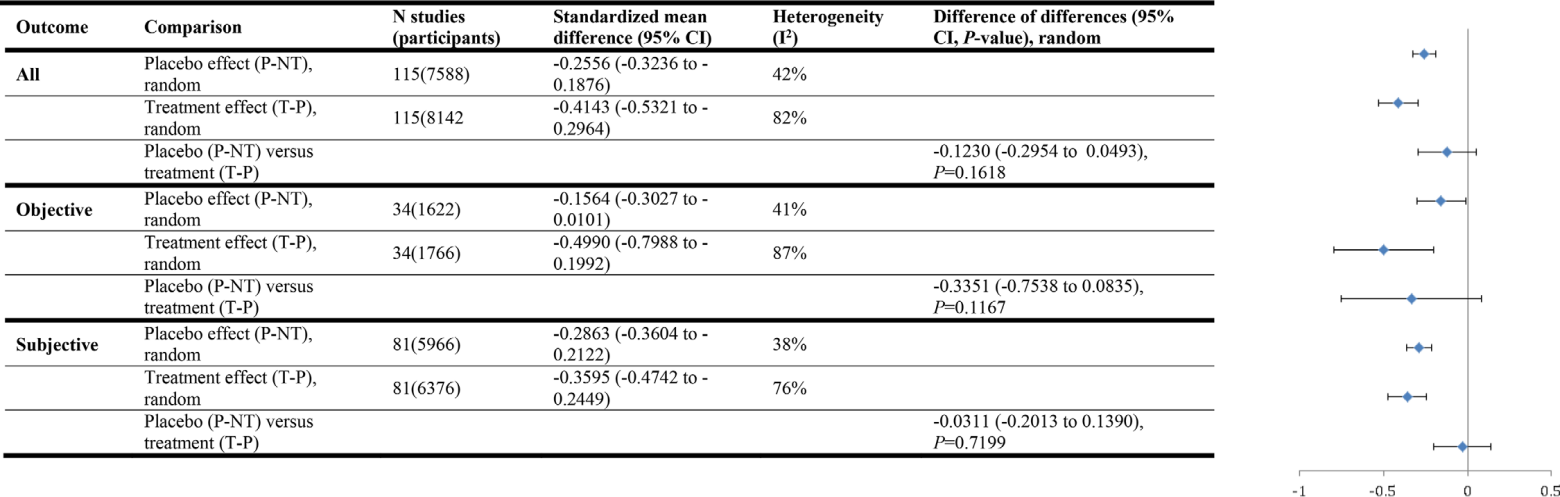
Placebo versus treatment effects (continuous outcomes)

**Fig 4:**
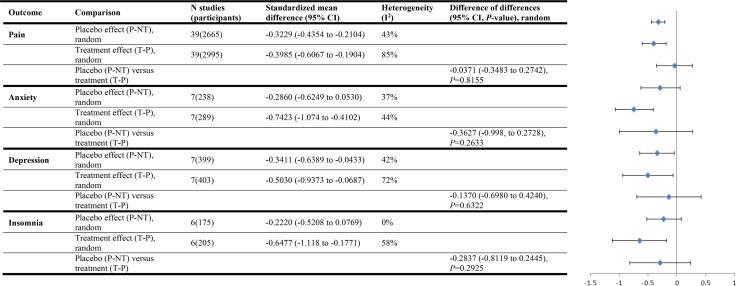
Conditions tested in three or more trials (continuous outcomes)

**Fig 5:**
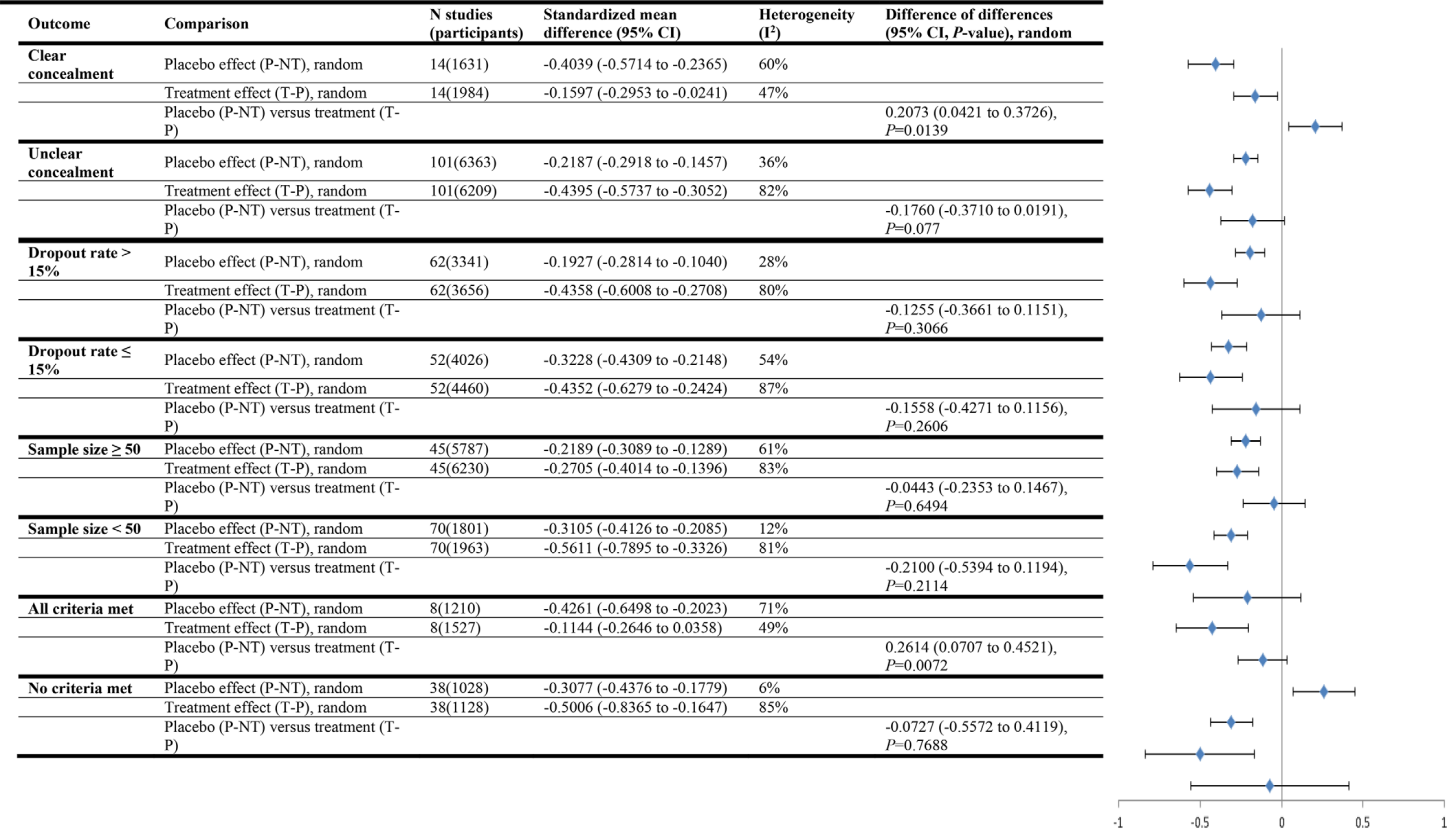
Trials with varying degrees of bias (continuous outcomes)

Our corrected analysis revealed no statistically significant difference between placebo effect sizes and treatment effect sizes in all but two subgroup analyses, and in both of them placebo effects were larger. These were: all studies that reported clear allocation concealment, and studies where all criteria for bias ruled out (placebo effects larger). Since placebo effects appear to be relatively greater when bias is ruled out, our corrected conclusions also reduce the possibility that placebo effects are attributable to bias.

It is important to note that the placebo and treatment effects may not be independent. For example large placebo effects could be correlated with small treatment effects. Hence there is currently no perfect statistical solution to the problem of comparing effects of placebos and treatments within three-armed trials (no treatment, placebo, treatment). However using the within study difference in effects (placebo effect–treatment effect) and an estimate of the standard deviation of this comparison based on assuming (perhaps incorrectly) independence between effects, allows the use of the standardized mean difference to obtain a pooled estimate of the difference between these two effects.

Professor Stephen Senn assisted with the analysis that allowed us to recalculate the differences between placebo and treatment effects in this *Erratum*.
