# Supplementary material for: Are Treatments More Effective than Placebos? A Systematic Review and Meta-Analysis
Source: PLoS One. 2013 May 15;8(5):e62599. doi: 10.1371/journal.pone.0062599 (PMC3655171; doi:10.1371/journal.pone.0062599)
Supplement: Appendix S2 — Hypothesis test for binary outcomes. (DOCX) [file pone.0062599.s002.docx]

**Appendix 2. Hypothesis test for binary outcomes**

Denote the proportion of events in the Treatment, Placebo, and No-Treatment group as:

$$P_{t}= \frac{d_{t}}{n_{t}}, P_{p} = \frac{d_{p}}{n_{p}}, P_{nt}= \frac{d_{nt}}{n_{nt}} .$$

The relevant Risk Ratios for the Treatment and Placebo group can be expressed as:

${RR}_{t}= \frac{P_{t}}{P_{p}}$ , ${RR}_{p}= \frac{P_{p}}{P_{nt}}$ .

We can then formulate the null and alternative Hypothesis for equivalence in the Treatment and Placebo effect as:

Ho: $\frac{RR_{t}}{RR_{p}}$ = 1 , Ha: $\frac{RR_{t}}{RR_{p}}$ ≠ 1 .

A point estimate for this statistic can be based on:

$\frac{\tilde{RRp}}{\tilde{RRp}}=$ $\frac{\frac{\tilde{P_{t}}}{\tilde{P_{p}}}}{\frac{\tilde{P_{p}}}{\tilde{P_{nt}}}}= \frac{\check{P_{t}} \tilde{P_{nt}}}{\tilde{P_{p}^{2}}}$

with variance: Var $\left\lfloor\frac{\tilde{P_{t}} \tilde{P_{nt}}}{\tilde{P_{p}^{2}}} \right\rfloor$ .

Using the same technique as for calculating 95% for the relative risk (RR), define the 95% CI as:

$\frac{\frac{\tilde{{RR}_{r}}}{\tilde{{RR}_{p}}}}{{EF}_{tp}}$ to $\frac{\tilde{{RR}_{t}}}{\tilde{{RR}_{p}}} x {EF}_{tp}$, where:

${EF}_{tp}=\exp\left\lfloor1.96 x SE (log\left( \frac{\tilde{{RR}_{r}}}{\tilde{{RR}_{p}}} \right)) \right\rfloor$, with

SE $\left\lfloor\log\left( \tilde{\frac{{RR}_{T}}{\tilde{{RR}_{p}}}} \right) \right\rfloor=SE \left\lfloor\log\left( \tilde{P_{T}} \right)+\log\left( \tilde{P_{NT}} \right)-2\log\left( \tilde{P_{p}} \right) \right\rfloor$, and

$SE\left\lfloor. \right\rfloor= \sqrt{V \left\lfloor. \right\rfloor}$ , or ${SE}^{2}\left\lfloor. \right\rfloor=V \left\lfloor. \right\rfloor$ .

Hence: $V \left\lfloor\log\tilde{P_{t}} \right\rfloor+\log\left( \tilde{P_{nt}} \right)-2\log\left( \tilde{P_{p}} \right)=$

$V \left\lfloor\log\left( \tilde{P_{T}} \right) \right\rfloor+V \left\lfloor\log\left( \tilde{P_{NT}} \right) \right\rfloor+4 V \left( \log\left( \check{P_{p}} \right) \right)+ \sum Cov\left( . \right)$ ,

with $\sum Cov\left( . \right)=0$, which gives

$V\left\lfloor\log\left( \tilde{\frac{{RR}_{T}}{\tilde{{RR}_{p}}}} \right) \right\rfloor=\left\lfloor\frac{1}{d_{t}}- \frac{1}{n_{t}} \right\rfloor+ \left\lfloor\frac{1}{d_{nt}}- \frac{1}{n_{nt}} \right\rfloor+4 \left\lfloor\frac{1}{d_{p}}- \frac{1}{n_{p}} \right\rfloor$.
