# Supplementary material for: Are Treatments More Effective than Placebos? A Systematic Review and Meta-Analysis
Source: PLoS One. 2013 May 15;8(5):e62599. doi: 10.1371/journal.pone.0062599 (PMC3655171; doi:10.1371/journal.pone.0062599)
Supplement: Appendix S3 — References to studies included in this review. (DOCX) [file pone.0062599.s003.docx]

**Appendix 3. References to studies included in this review**

**Abikoff 2004 *{published data only}***

Abikoff H, Hechtman L, Klein RG, Gallagher R, Fleiss K, Etcovitch J, et al. Social functioning in children with ADHD treated with long-term methylphenidate and multimodal psychosocial treatment. *Journal of the American Academy of* *Child and Adolescent Psychiatry* 2004; **43**: 820–9.

**Adriaanse 1995 *{published data only}***

Adriaanse AH, Kollée LAA, Muytjens HL, Nijhuis JG, de Haan AFJ, Eskes TKAB. Randomized study of vaginal chlorhexidine disinfection during labor to prevent vertical transmission of group B streptococci. *European Journal of* *Obstetrics, Gynecology and Reproductive Biology* 1995; **61**: 135–41. [MEDLINE: 7556834]

**Alfano 2001a, b**

Alfano AP, Taylor AG, Foresman PA, Dunkl PR,McConnell GG, Conaway MR, Gillies GT. Static magnetic fields for treatment of fibromyalgia: a randomised controlled trial. *Journal of Alternative and Complementary Medicine* 2001;**7**: 53–64.

**Alkaissi 1999 *{published data only}***

Alkaissi A, Stålnert M, Kalman S. Effect and placebo effect of acupressure (P6) on nausea and vomiting after outpatientgynaecological surgery. *Acta anaesthesiologica Scandinavica* 1999; **43**: 270–4.

**Alkaissi 2002 *{published data only}***

Alkaissi A, Evartsson K, Johnsson VA, Ofenbartl L, Kalman S. P6 acupressure may relieve nausea and vomiting after gynecological surgery: an effectiveness study in 410 women. *Canadian Journal of Anaesthesia* 2002; **49**: 1034–9.

**Allen 1998 *{published data only}***

Allen JJB, Schnyder RN, Hitt SK. The efficacy of acupuncture in the treatment of major depression in women. *Psychological Science* 1998; **9**:397–401.

**Allen 2006 *{published data only}***

Allen JJB, Schnyer RN, Chambers AS, Hitt SK, Moreno FA, Manber R. Acupuncture for depression: a randomized

controlled trial. *Journal of Clinical Psychiatry* 2006; **67**: 1665–73.

**Antonio 1999 *{published data only}***

Antonio J, Colker CM, Torina G, Shi Q, Brink W, Kalman D. Effects of standardised guggulsterone phosphate supplement on body composition in overweight adults: a pilot study. *Current Therapeutic Research* 1999; **60**: 220–7.

**Ascher 1979 *{published data only}***

Ascher LM, Turner RM. Paradoxical intention and insomnia: an experimental investigation. *Behaviour Research* *and Therapy.* 1979; **17**:408–11. [MEDLINE: 526243]

**Aune 1998 *{published data only}***

Aune A, Alraek T, Lihua H, Baerheim A. Acupuncture in the prophylaxis of recurrent lower urinary tract infection in adult women. *Scandinavian Journal of Primary Health Care* 1998; **16**: 37–9.

**Benedetti 1995 *{published data only}***

Benedetti F, Amanzio M, Maggi G. Potentiation of placebo analgesia by proglumide. *Lancet* 1995; **346**: 1231. [MEDLINE: 7475687]

**Berg 1983 *{published data only}***

Berg I, Forsythe I, Holt P, Watts J. A controlled trial of ’senokot’ in faecal soiling treated by behavioural methods.

*Journal of Child Psychology and Psychiatry and Allied Disciplines* 1983; **24**(4): 543–9. [MEDLINE: 6630328]

**Blackman 1964 *{published data only}***

Blackman S, Benton AJ, Cove LM. The effect of imipramine on enuresis. *The American Journal of Psychiatry* 1964; **120**: 1194–5.

**Blanchard 1990a *{published data only}***

Blanchard EB, Appelbaum KA, Radnitz CL, Michultka D, et al.Placebo-controlled evaluation of abbreviated progressive muscle relaxation and of relaxation combined with cognitive therapy in the treatment of tension headache. *Journal of Consulting and Clinical Psychology* 1990; **58**: 210–5.

**Blanchard 1990b *{published data only}***

Blanchard EB, Appelbaum KA, Radnitz CL, Morrill B, et al. A controlled evaluation of the thermal biofeedback and thermal biofeedback combined with cognitive therapy in the treatment of vascular headache. *Journal of Consulting* *and Clinical Psychology* 1990; **58**: 216–24.

**Block 1980 *{published data only}***

Block J. Effects of rational emotive therapy on overweight adults. *Psychotherapy: Theory, Research and Practice* 1980; **17** (3): 277–80.

**Bosley 1989 *{published data only}***

Bosley F, Allen TW. Stress management training for hypertensives: cognitive and physiological effects. *Journal* *of Behavioral Medicine* 1989; **12**(1): 77–89. [MEDLINE: 2746644]

**Bramston 1985 *{published data only}***

Bramston P, Spence SH. Behavioural versus cognitive social-skills training with intellectually-handicapped adults. *Behaviour Research and Therapy* 1985; **23**(3): 239–46. [MEDLINE: 4004703]

**Brinkhaus 2006 *{published data only}***

Brinkhaus B, Witt CM, Jena S, Linde K, Streng A, Wagenpfeil S, et al. Acupuncture in patients with chronic low back pain: a randomized controlled trial. *Arch Intern Med* 2006; **166**: 450–7.

**Cabrini 2006 *{published data only}***

Cabrini L, Gioia L, Gemma M, Melloni G, Caretta A, Ciriaco P, Puglisi A. Acupuncture for diagnostic bronchoscopy: a prospctive, randomized, placebocontrolled study. *American Journal of Chinese Medicine* 2006; **34**: 409–15.

**Carter 2003 *{published data only}***

Carter JC, Olmsted MP, Kaplan AS, McCabe RE, Mills JS, Aime A. Self-help for bulimia nervosa: a randomized controlled trial. *American Journal of Psychiatry* 2003; **160**: 973–8.

**Classen 1983 *{published data only}***

Classen W, Feingold E, Netter P. Influence of sensory suggestibility on treatment outcome in headache patients. *Neuropsychobiology* 1983; **10**: 44–7. [MEDLINE: 6657038]

**Colker 1999 *{published data only}***

Colker CM, Kalman DS, Torina GC, Perlis T, Street C. Effects of Citrus aurantium extract, caffeine, and St. John’s wort on body fat loss, lipid levels, and mood states in overweight healthy adults. *Current Therapeutic Research* 1999; **60**: 145–53.

**Conn 1986 *{published data only}***

Conn IG, Marshall AH, Yadev SN, Daly J, Jaffer M. Transcutaneous electrical nerve stimulation following appendicectomy: the placebo effect. *Annals of the Royal* *College of Surgeons of England* 1986; **68**: 191–2. [MEDLINE: 3538985]

**Costello 2006 *{published data only}***

Costello M, Ramundo M, Christopher NC, Powel KR. Etyhyl vinyl chloride vapocoolant spray fails to decrease pain associated with intravenous cannulation in children. *Clinical Pediatrics* 2006; **45**: 628–32.

**Crosby 1994 *{published data only}***

Crosby L, Palarski VA, Cottington E, Cmolik B. Iron supplementation for acute blood loss anemia after coronary artery bypass surgery: a randomized, placebo-controlled study. *Heart & Lung* 1994; **23**: 493–9. [MEDLINE: 7852064]

**Cupal 2001 *{published data only}***

Cupal DD, Brewer BW. Effects of relaxation and guided imagery on knee strength, reinjury anxiety, and pain following anterior cruciate ligament reconstruction. *Rehabilitation Psychology* 2001; **46**: 28–43.

**Davidson 1980 *{published data only}***

Davidson A, Denney DR, Elliott CH. Suppression and substitution in the treatment of nailbiting. *Behaviour* *Research and Therapy* 1980; **18**: 1–9. [MEDLINE: 7369982]

**Defrin 2005**

Defrin R, Ariel E, Peretz C. Segmental noxious versus innocuous electrical stimulation for chronic pain relief and the effect of fading sensation during treatment. *Pain* 2005; **115**:152–60.

**Ditto 2003 *{published data only}***

Ditto B, France CR, Lavoie P, Roussos M, Adler PS. Reducing reactions to blood donation with applied muscle tension: a randomized controlled trial. *Transfusion* 2003; **43**: 1269–75.

**Ditto 2006 *{published data only}***

Ditto B, France CR. The effects of applied tension on symptoms in French-speaking blood donors: a randomized trial. *Health Psychology* 2006; **25**: 433–7.

**Double 1993 *{published data only}***

Double DB, Warren GC, Evans M, Rowlands MP. Efficacy of maintenance use of anticholinergic agents. *Acta*

*Psychiatrica Scandinavica* 1993; **88**: 381–4. [MEDLINE: 7905226]

**Dundee 1986 *{published data only}***

Dundee JW. Belfast experience with P6 acupuncture antiemesis. *The Ulster Medical Journal* 1990; **59**(1):63–70.

[MEDLINE: 2349751]_ Dundee JW, Chestnutt WN, Ghaly RG, Lynas AGA. Traditional Chinese acupuncture: a potentially useful antiemetic? *British Medical Journal* 1986; **293**: 583–4. [MEDLINE: 3092933]

**Elliott 1978 *{published and unpublished data}***

Elliott CH, Denney DR. A multiple-component treatment approach to smoking reduction. *Journal of Consulting and Clinical Psychology* 1978; **46**(6): 1330–9. [MEDLINE: 730881]

**Erdogmus 2007 *{published data only}***

Erdogmus CB, Resch KL, Sabitzer R, Müller H, Nuhr M, Schöggl A, et al. Physiotherapy-based rehabilitation following disc herniation operation. *Spine* 2007; **32**: 2041–9.

**Espie 1989 *{published data only}***

Espie CA, Lindsay WR, Brooks DN, Hood EM, Turvey T. A controlled comparative investigation of psychological treatments for chronic sleep-onset insomnia. *Behaviour* *Research and Therapy* 1989; **27**(1): 79–88. [MEDLINE: 2914008]

**Etringer 1982 *{published data only}***

Etringer BD, Cash TF, Rimm DC. Behavioral, affective and cognitive effects of participant modeling and an equally credible placebo. *Behavior Therapy* 1982; **13**: 476–85.

**Etter 2002 *{published data only}***

Etter J, Lazlo E, Zellweger J, Perrot C, Perneger TV. Nicotine replacement to reduce cigarette consumption in smokers who are unwilling to quit: a randomized trial. *Journal of Clinical Psychopharmacology* 2002; **22**: 487–95.

**Faas 1995 *{published data only}***

Faas A, van Eijk JTM, Chavannes AW, Gubbels JW. A randomized trial of exercise therapy in patients with acute low back pain. Efficacy on sickness absence. *Spine* 1995; **20** (8): 941–7. [MEDLINE: 7644960]

**Fanti 2003 *{published data only}***

Fanti L, Gemma M, Passaretti S, Guslandi M, Testoni PA, Casti A, Torri G. Electroacupuncture analgesia for colonoscopy: a prospective, randomised, placebo-controlled study. *The American Journal of Gastroenterology* 2003; **98**: 312–6.

**Fisher 2006 *{published data only}***

Fisher P, McCarney R, Hasford C, Vickers A. Evaluation of specific and non-specific effects in homeopathy: feasibility study for a randomised trial. *Homeopath* 2006; **95**: 215–22.

**Forster 1994 *{published data only}***

Forster EL, Kramer JF, Lucy SD, Scudds RA, et al. Effects of TENS on pain, medications, and pulmonary function following coronary artery bypass graft surgery. *Chest* 1994; **106**(5): 1343–8. [MEDLINE: 7956382]

**Foster 2004 *{published data only}***

Foster KA, Liskin J, Cen S, Abbot A, Armisen V, Globe D, et al. The Trager approach in the treatment of chronic headache: a pilot study. *Alternative Therapies in Health andMedicine* 2004; **10**: 40–6.

**Foster 2007 *{published data only}***

Foster NE, Thomas E, Barlas P, Hill JC, Young J, Mason E, Hay EM. Acupuncture as an adjunct to excercise based physiotherapy for osteoarthritis of the knee: randomized controlled trial. *BMJ* 2007; **335**: 436.

**Frank 1990 *{published data only}***

Frank E, Kupfer DJ. Does a placebo tablet affect psychotherapeutic treatment outcome? Results from the Pittsburgh study of maintenance therapies in recurrent depression. *Psychotherapy Research* 1992; **2**(2): 102–11.

**Frankel 1978 *{published data only}***

Frankel BL, Patel DJ, Horwitz D, Friedewald WT, et al. Treatment of hypertension with biofeedback and relaxation techniques. *Psychosomatic Medicine* 1978; **40**(4): 276–93. [MEDLINE: 356078]

**Fuchs 1977 *{published data only}***

Fuchs CZ, Rehm LP. A self-control behavior therapy program for depression. *Journal of Consulting and Clinical* *Psychology* 1977; **45**(2): 206–15. [MEDLINE: 850005]

**Goodenough 1997 *{published data only}***

Goodenough B, Kampel L, Champion GD, Laubreaux L, Nicholas K, Ziegler JB, et al.An investigation of the placebo effect and age-related factors in the report of needle pain from venipuncture in children. *Pain* 1997; **72**: 383–91. [MEDLINE: 9313279]

**Guglielmi 1982 *{published data only}***

Guglielmi RS, Roberts AH, Patterson R. Skin temperature biofeedback for Raynaud’s disease: a double-blind study. *Biofeedback and Self-Regulation* 1982; **7**(1): 99–120. [MEDLINE: 7093357]

**Hall 1974 *{published data only}***

Hall SM, Hall RG, Hanson RW, Borden BL. Permanence of two self-managed treatments of overweight in university and community populations. *Journal of Consulting and Clinical* *Psychology* 1974; **42**(6): 781–6. [MEDLINE: 4436463]

**Hanson 1976 *{published data only}***

Hanson RW, Borden BL, Hall SM. Use of programmed instruction in teaching self-management skills to overweight adults. *Behavior Therapy* 1976; **7**(3): 366–73.

**Hargreaves 1989 *{published data only}***

Hargreaves A, Lander J. Use of transcutaneous electrical nerve stimulation for postoperative pain. *Nursing Research* 1989; **38**(3): 159–61. [MEDLINE: 2785684]

**Harrison 1975 *{published data only}***

Harrison RF, de Louvois J, BladesM, Hurley R. Doxycycline treatment and human infertility. *Lancet* 1975; **i**(7907): 605–7. [MEDLINE: 47949]

**Hashish 1986 *{published data only}***

Hashish I, Harvey W, Harris M. Anti-inflammatory effects of ultrasound therapy: evidence for a major placebo effect. *British Journal of Rheumatology* 1986; **25**: 77–81. [MEDLINE: 2417648]

**Hashish 1988 *{published data only}***

Hashish I, Hai HK, Harvey W, Feinmann C, Harris M. Reduction of postoperative pain and swelling by ultrasound treatment: a placebo effect. *Pain* 1988; **33**: 303–11. [MEDLINE: 3419838]

**Hawkins 1995**

Hawkins PJ, Liossi C, Ewart BW, Hatira P, Kosmidis VH, Varvutsi M. Hypnotherapy for control of anticipatory nausea and vomiting in children with cancer: preliminary findings. *Psycho-Oncology* 1995;**4**:101–6.

**Heinzl**

Heinzl S, Andor J. Preoperative administration of prostaglandin to avoid dilatation-induced damage in firsttrimester

pregnancy terminations. *Gynecologic and Obstetric Investigation* 1981;**12**:29–36. [MEDLINE: 7250779]

**Helms 1987 *{published data only}***

Helms JM. Acupuncture for the management of primary dysmenorrhea. *Obstetrics & Gynecology* 1987; **69**(1): 51–6. [MEDLINE: 3540764]

**Hong 1993 *{published data only}***

Hong C, Chen Y, Pon CH, Yu J. Immediate effects of various physical medicine modalities on pain threshold of an active myofascial trigger point. *Journal of Muscoloskeletal* *Pain* 1993; **1**(2): 37–53.

**Hovell 2003**

Hovell MF, Sipan CL, Blumberg EJ, Hofstetter CR, Slymen D, Friedman L, et al.Increasing Latino adolescents’ adherence to treatment for latent tuberculosis infection: a controlled trial. *American Journal of Public Health* 2003;**93**: 1871–7.

**Hruby 2006 *{published data only}***

Hruby G, Ames C, Chen C, Yan Y, Sagar J, Baron P, Landman J. Assessment of efficacy of transcutaneous electrical nerve stimulation for pain management during office-based flexible cystoscopy. *Urology* 2006; **67**: 914–7.

**Hutton 1991 *{published data only}***

Hutton N, Wilson MH, Mellits D, Baumgardner R, et al. Effectiveness of an antihistamine-decongestant combination for young children with the common cold: a randomized, controlled clinical trial. *The Journal of* *Pediatrics* 1991; **118**(1): 125–30. [MEDLINE: 1670783]

**Hyland 2006 *{published data only}***

Hyland MR, Webber-Gaffney A, Cohen L, Lichtman SW. Randomized controlled trial of calcaneal taping, sham taping, and plantar fascia stretching for the short-term management of plantar heel pain. *Journal of Orthopaedic* *and Sports Physical Therapy* 2006; **36**: 364–71.

**Hyman 1986 *{published data only}***

Hyman GJ, Stanley RO, Burrows GD,Horne DJ. Treatment effectiveness of hypnosis and behaviour therapy in smoking cessation: a methodological refinement. *Addictive Behaviors* 1986; **11**: 355–65. [MEDLINE: 3812046]

**Jacobs 1971 *{published data only}***

Jacobs MA, Spilken AZ, Norman MM, Wohlberg GW, Knapp PH. Interaction of personality and treatment conditions associated with success in a smoking control program. *Psychosomatic Medicine* 1971; **33**(6): 545–56. [MEDLINE: 5148984]

**Jacobson 1978 *{published data only}***

Jacobson NS. Specific and nonspecific factors in the effectiveness of a behavioral approach to the treatment of marital discord. *Journal of Consulting and Clinical Psychology* 1978; **46**(3): 442–52. [MEDLINE: 670488]

**Kaptchuk 2008 *{published data only}***

Kaptchuk TJ, Kelley JM, Conboy LA, Davis RB, Kerr CE, Jacobsen EE, et al. Components of placebo effect: randomised controlled trial in patients with irritable bowel syndrome. *BMJ* 2008; **online**: 1–8.

**Karst 2007 *{published data only}***

Karst M, Wintherhalter M, Münte S, Francki B, Hondronikos A, Eckardt A, et al. Auricular acupuncture for dental anxiety: a randomized controlled trial. *Anesthesia &* *Analgesia* 2007; **104**: 295–300.

**Karunakaran 1997 *{published and unpublished data}***

Karunakaran S, Hammersley MS, Morris RC, Turner RC, et al.The fasting hyperglycaemia study: III. Randomized controlled trial of sulfonylurea therapy in subjects with increased but not diabetic fasting plasma glucose. *Metabolism* 1997; **46**(12,suppl 1): 56–60. [MEDLINE: 9439561]

**Kendall 1979 *{published data only}***

Kendall PC, Williams L, Pechacek TF, Graham LE, Shisslak C, Herzoff N. Cognitive-behavioral and patient education interventions in cardiac catheterization procedures: the Palo Alto medical psychology project. *Journal of Consulting* *and Clinical Psychology* 1979; **47**(1): 49–58. [MEDLINE: 429666]

**Kerr 2003 *{published data only}***

Kerr AR, Drexel CA, Spielman AI. The efficacy and safety of 50 mg penicillin G potassium troches for recurrent aphthous ulcers. *Oral Surgery, Oral Medicine, Oral Pathology, Oral Radiology & Endodontics* 2003; **96**: 685–94.

**Killeen 2004 *{published data only}***

Killeen TK, Brady KT, Gold PB, Simpson KN, Faldowski RA, Tyson C, Anton RF. Effectiveness of naltrexone in a community treatment program. *Alcoholism, Clinical and Experimental Research* 2004; **28**: 1710–7.

**Killen 1990 *{published data only}***

Killen JD, Fortmann SP, Newman B, Vardy A. Evaluation of a treatment approach combining nicotine gum with self-guided behavioural treatments for smoking relapse prevention. *Journal of Consulting and Clinical Psychology* 1990; **58**(1): 85–92. [MEDLINE: 2319049]

**Kilmann 1987 *{published data only}***

Kilman PR, Milan RJ, Boland JP, Nankin HR, Davidson E, West MO, et al. Group treatment of secondary erectile dysfunction. *Journal of Sex & Marital Therapy* 1987; **13**: 168–82.

**Klerman 1974 *{published data only}***

Klerman GL, DiMascio A, Weisman M, Prusoff B. Treatment of depression by drugs and psychotherapy. *The American Journal of Psychiatry* 1974; **131**(2): 186–91. [MEDLINE: 4587807]

**Kober 2002 *{published data only}***

Kober A, Scheck T, Greher M, Lieba F, Fleischhackl R, Fleischhackl S, et al. Prehospital analgesia with acupressure in victims of minor trauma: a prospective, randomized, double-blinded trial. *Anesthesia and Analgesia* 2002; **95**: 723–7.

**Kotani 2001 *{published data only}***

Kotani N, Kushikata T, Suzuki A, Hashimoto H, Muraoka M, Matsuki A. Insertion of intradermal needles into painful points provides analgesia for intractable abdominal scar pain. *Regional Anesthesia and Pain Medicine* 2001; **26**: 532–8.

**Lander 1993 *{published data only}***

Lander J, Fowler-Kerry S. TENS for children’s procedural pain. *Pain* 1993; **52**: 209–16. [MEDLINE: 8455969]

**Leibing 2002 *{published data only}***

Leibing E, Leonhardt U, Köster G, Goerlitz A, Rosenfeldt JA, Hilgers R, Ramadori G. Acupuncture treatment of chronic low-back pain: a randomized, blinded, placebocontrolled trial with 9-months follow-up. *Pain* 2002; **96**: 189–96.

**Lick 1975 *{published data only}***

Lick J. Expectancy, false galvanic skin response feedback and systematic desensitization in the modification of phobic behavior. *Journal of Consulting and Clinical Psychology* 1975; **43**(4): 557–67. [MEDLINE: 239972]

**Lick 1977 *{published data only}***

Lick JR, Heffler D. Relaxation training and attention placebo in the treatment of severe insomnia. *Journal of* *Consulting and Clinical Psychology* 1977; **45**(2): 153–61. [MEDLINE: 321491]

**Limoges 2004 *{published data only}***

Limoges MF, Rickabaugh B. Evaluation of TENS during screening flexible sigmoidoscopy. *Gastroenterol Nurs* 2004; **27**: 61–8.

**Lin 2002 *{published data only}***

Lin JG, Lo MW, Wen YR, Hsieh CL, Tsai SK, Sun WZ. The effect of high and low frequency electroacupuncture in pain after lower abdominal surgery. *Pain* 2002; **99**: 509–14.

**Linde 2005 *{published data only}***

Linde K, Streng A, Jurgens S, Hoppe A, Brinkhaus B, Witt C, et al. Acupuncture for patients with migraine: a randomized controlled trial. *JAMA* 2005; **293**: 2118–25.

**Lindholm 1996 *{published data only}***

Lindholm LH, Ekbom T, Dash C, Isacsson Å, et al. Changes in cardiovascular risk factors by combined pharmacological and nonpharmacological strategies: the main result of the CELL study. *Journal of Internal Medicine* 1996; **240**: 13–22. [MEDLINE: 8708586]

**Liossi 2003 *{published data only}***

Liossi C, Hatira P. Clinical hypnosis in the alleviation of procedure-related pain in pediatric oncology patients. *International Journal of Clinical and Experimental Hypnosis* 2003; **51**: 4–28.

**Longo 1988 *{published data only}***

Longo DJ, Clum GA, Yaeger NJ. Psychosocial treatment for recurrent genital herpes. *Journal of Consulting and Clinical* *Psychology* 1988; **56**(1): 61–6. [MEDLINE: 3279091]

**Macaluso 1995 *{published data only}***

Macaluso AD, Conelly AM, Hayes WB, Houb MC, et al. Oral transmucosal fentanyl citrate for premedication in adults. *Anesthesia and Analgesia* 1996; **82**: 158–61. [MEDLINE: 8712394]

**Malcolm 1980 *{published data only}***

Malcolm RE, Sillet RW, Turner JAM, Ball KP. The use of nicotine chewing gum as an aid to stopping smoking. *Psychopharmacology* 1980; **70**: 295–6. [MEDLINE: 6777804]

**Markland 1993 *{published data only}***

Markland D, Hardy L. Anxiety, relaxation and anaesthesia for day-case surgery. *British Journal of Clinical Psychology* 1993; **32**: 493–504. [MEDLINE: 8298547]

**Matros 2006 *{published data only}***

Matros E, Rocha F, Zinner M, Wang J, Ashley S, Breen E, et al. Does gum chewing ameliorate postoperative ileus? Results of a prospective, randomized, placebo-controlled trial. *Journal of the American College of Surgeons* 2006; **202**: 773–8.

**McMillan 1994 *{published and unpublished data}***

McMillan CM. Transcutaneous electrical stimulation of Neiguan anti-emetic acupuncture point in controlling sickness following opioid analgesia in major orthopaedic surgery. *Physiotherapy* 1994; **80**(1):5–9.

**Medici 2002 *{published data only}***

Medici TC, Grebski E, Wu J, Hinz G, Wuthrich B. Acupuncture and bronchial asthma: a long-term randomized study of the effects of real versus sham acupuncture compared with controls in patients with bronchial asthma. *Journal of Alternative and Complementary* *Medicine* 2002; **8**: 737–50.

**Melchart 2005 *{published data only}***

Melchart D, Streng A, Hoppe A, Brinkhaus B, Witt C, Wagenpfeil S, et al. Acupuncture in patients with tensiontype headache: randomised controlled trial. *BMJ* 2005; **331**:376–82.

**Moffet 1996 *{published data only}***

Moffett JAK, Richardson PH, FrostH, Osborn A. A placebo controlled double blind trial to evaluate the effectiveness of pulsed short wave therapy for osteoarthritic hip and knee pain. *Pain* 1996; **67**: 121–7. [MEDLINE: 8895239]

**Molsberger 2002 *{published data only}***

Molsberger AF, Mau J, Pawelec DB, Winkler J. Does acupuncture improve the orthopedic management of chronic low back pain: a randomized, blinded, controlled trial with 3 months follow up. *Pain* 2002; **99**: 579–87.

**Moreland 2006 *{published data only}***

Moreland EC, Volkening LK, Lawlor MT, Chalmers KA, Anderson BJ, Laffel LM. Use of a blood glucose monitoring manual to enhance monitoring adherence in adults with diabetes: a randomized controlled trial. *Archives of Internal* *Medicine* 2006; **166**: 689–95.

**Morey 2006 *{published data only}***

Morey MC, Ekelund C, Pearson M, Crowley G, Peterson M, et al.Project LIFE: a partnership to increase physical activity in elders with multiple chronic illnesses. *J Aging Physical Act* 2006; **14**: 324–43.

**Murphy 1982 *{published data only}***

Murphy JK, Williamson DA, Buxton AE, Moody SC, et al. The long-term effects of spouse involvement upon weight loss and maintenance. *Behavior Therapy* 1982; **13**: 681–93.

**Najnigier 1997 *{published data only}***

Najnigier B, Patkowski W, Zieniewicz K, Nyckowski P, et al. Zofran (ondansetron) in preventing postoperative nausea and vomiting after laparoscopic cholecystectomy [Zofran w zapobieganiu nudnosciom i wymiotom po cholecystektomii laparoskopowej]. *Acta Endoscopica Polona* 1997; **7**(3): 125–8.

**Nandi 1976 *{published data only}***

Nandi DN, Ajmany S, Ganguli H, Banerjee G, et al. A clinical evaluation of depressives found in a rural survey in

India. *The British Journal of Psychiatry; the Journal of Mental Science* 1976; **128**: 523–7. [MEDLINE: 1276560]

**Nicassio 1974 *{published data only}***

Nicassio P, Bootzin R. A comparison of progressive relaxation and autogenic training as treatments for insomnia. *Journal of Abnormal Psychology* 1974; **83**(3): 253–60. [MEDLINE: 4844912]

**Nocella 1982 *{published data only}***

Nocella J, Kaplan RB. Training children to cope with dental treatment. *Journal of Pediatric Psychology* 1982; **7**(2): 175–8. [MEDLINE: 6125579]

**Parker 2003 *{published data only}***

Parker JC, Smarr KL, Slaughter JR, Johnston SK, Priesmeyer ML, Hanson KD, et al. Management of depression in rheumatoid arthritis: a combined pharmacologic and cognitive-behavioral approach. *Arthritis and Rheumatism* 2003; **49**: 766–77.

**Pelham 1992 *{published data only}***

Pelham WE, Murphy DA, Vannatta K, Milich R, et al. Methylphenidate and attributions in boys with attentiondeficit hyperactivity disorder. *Journal of Consulting and Clinical Psychology* 1992; **60**(2): 282–92. [MEDLINE: 1592959]

**Quahagen 1995**

Quayhagen MP, Quayhagen M, Corbeil RR, Roth P, et al. A dyadic remediation program for care recipients

with dementia. *Nursing Research* 1995;**44**(3):153–9. [MEDLINE: 7761291]

**Rawling 2001 *{published data only}***

Rawling MJ, Wiebe ER. A randomized controlled trial of fentanyl for abortion pain. *American Journal of Obstetrics* *and Gynecology* 2001; **185**: 103–7.

**Reading 1982 *{published data only}***

Reading AE. The effects of psychological preparation on pain and recovery after minor gynaecological surgery: a preliminary report. *Journal of Clinical Psychology* 1982; **38** (3): 504–12. [MEDLINE: 6213641]

**Ristikankare 1999 *{published data only}***

Ristikankare M, Hartikainen, Heikkinen M, Janatuinen E, Julkunen R. Is routinely given conscious sedation of benefit during colonoscopy?. *Gastroenterology Endoscopy* 1999; **49**: 566–72.

**Robinson 2001 *{published data only}***

Robinson R, Darlow S, Wright SJ, Watters C, Carr I, Gadsby G, Mayberry J. Is transcutaneous electrical nerve stimulation an effective analgesia during colonoscopy. *Postgraduate Medical Journal* 2001; **77**:445–6.

**Roongpisuth 1999**

Roongpisuthipong C, Panpakdee O, Boontawee A, Kulapongse S, Tanphaichitr V. Possible thermogenesis with dexfenfluramine. *Chotmaihet thangphaet [Journal of the Medical Association of Thailand]* 1999;**82**:150–9.

**Roscoe 2002 *{published data only}***

Roscoe JA, Morrow GR, Bushunow P, Tian L, Matteson S. Acustimulation wristbands for the relief of chemotherapyinduced nausea. *Alternative Therapies* 2002; **8**: 56–62.

**Roscoe 2005 *{published data only}***

Roscoe JA, Matteson SE, Morrow GR, Hickok JT, Bushunow P, Griggs J, et al.Acustimulation wrist bands are not effective for the control of chemotherapy-induced nausea in women with breast cancer. *Journal of Pain and* *Symptom Management* 2005; **29**: 376–84.

**Rosen 1976 *{published data only}***

Rosen GM, Glasgow RE, Barrera M. A controlled study to assess the clinical efficacy of totally self-administrated systematic desensitization. *Journal of Consulting and Clinical* *Psychology* 1976; **44**(2): 208–17. [MEDLINE: 3523]

**Rossi 1982 *{published data only}***

Rossi A, Ziacchi V, Lomanto B. The hypotensive effect of a single daily dose of labetalol: a preliminary study. *International Journal of Clinical Pharmacology, Therapy and* *Toxicology* 1982; **20**(9): 438–45. [MEDLINE: 6754634]

**Roughan 1981 *{published data only}***

Roughan PA, Kunst L. Do pelvic floor excercises really improve orgasmic potential? *Journal of Sex and Marital* *Therapy* 1981; **7**(3): 223–8. [MEDLINE: 7345161]

**Rybarczyk 1990 *{published data only}***

Rybarczyk BD, Auerbach SM. Reminiscence interviews as stress management interventions for older patients undergoing surgery. *The Gerontologist* 1990; **30**(4): 522–8. [MEDLINE: 2394388]

**Sanders 1990 *{published data only}***

Sanders G, Tepe R, Maloney P, Reinert O. The effect of spinal manipulation on subjects with acute low back pain: a comparison of visual analog pain scores and serum beta endorphin levels. *Journal of Manipulative and Physiological* *Therapeutics* 1990; **13**(1): 58.

Sanders GE, Reinert O, Tepe R, Maloney P. Chiropractic adjustive manipulation on subjects with acute low back pain: visual analog pain scores and plasma beta-endorphin levels. *Journal of Manipulative and Physiological Therapeutics* 1990; **13**(7): 391–5. [MEDLINE: 2145384]

**Schallreuter 2002 *{published data only}***

Shallreuter KU, Moore J, Behrens-Williams S, Panske A, Harari M. Rapid initiation of repigmentation in vitiligo with dead sea climatotherapy in combination with pseudocatalase (PC-KUS). *International Journal of Dermatology* 2002; **41**: 482–7.

**Scharf 2006 *{published data only}***

Scharf HP, Mansmann U, Streitberger K, Witte S, Krämer J, Maier C, et al. Acupuncture and knee osteoarthritis: a three armed randomized trial. *Annals of Internal Medicine* 2006; **145**: 12–20.

**Scharff 2002 *{published data only}***

Scharff L, Marcus DA, Masek BJ. A controlled study of minimal-contact thermal biofeedback treatment in children with migraine. *Journal of Pediatric Psychology* 2002; **27**: 109–19.

**Seer 1980 *{published data only}***

Seer P, Raeburn JM. Meditation training and essential hypertension: a methodological study. *Journal of Behavioral* *Medicine* 1980; **3**(1): 59–71. [MEDLINE: 6995617]

**Senediak 1985 *{published data only}***

Senediak C, Spence SH. Rapid versus gradual scheduling of therapeutic contact in a family based behavioural weight control programme for children. *Behavioural Psychotherapy* 1985; **13**: 265–87.

**Shen 2000 *{published data only}***

Shen J, Wenger N, Glaspy J, Hays RD, Albert PS, Choi C, Shekelle PG. Electroacupuncture for control of myeloablative chemotherapy-induced emesis. *JAMA* 2000; **284**: 2755–61.

**Stabholz 1991 *{published data only}***

Stabholz A, Shapira J, Shur D, Friedman M, et al. Local application of sustained-release delivery system of chlorhexidine in Down’s syndrome population. *Clinical* *Preventive Dentistry* 1991; **13**(5):9–14. [MEDLINE: 1839722]

**Stewart 1991 *{published data only}***

Stewart JE, Jacobs-Schoen M, Padilla MR, Maeder LA, et al.The effect of cognitive behavioral intervention on oral hygiene. *Journal of Clinical Periodontology* 1991; **18**:219–22. [MEDLINE: 1856301]

**Stransky 1989 *{published data only}***

Stransky M, Rubin A, Lava NS, Lazaro RP. Treatment of carpal tunnel syndrome with vitamin B6: a double blind study. *Southern Medical Journal* 1989; **89**(7): 841–2.[MEDLINE: 2749352]

**Straub 2001 *{published data only}***

Straub WF, Spino MP, Alattar MM, Pfleger B, et al.The effect of chiropractic care on jet lag of Finnish junior elite athletes. *Journal of Manipulative and Physiological* *Therapeutics* 2001; **24**: 191–8.

**Sumaya 2001 *{published data only}***

Sumaya IC, Rienzi BM, Deegan JF, Moss DE. Bright light treatment decreases depression in institutionalized older adults: a placebo-controlled crossover study. *The Journals of* *Gerontology. Series A. Biological Sciences and Medical Sciences* 2001; **56A**: M356–60.

**Tan 1982 *{published data only}***

Tan SY, Poser EG. Acute pain in a clinical setting: effects of cognitive-behavioural skills training. *Behaviour Research* *and Therapy* 1982; **20**:535–45. [MEDLINE: 7159348]

**Tan 1986 *{published data only}***

Tan SY, Bruni J. Cognitive-behavior therapy with adult patients with epilepsy: a controlled outcome study. *Epilepsia* 1986; **27**(3):225–32. [MEDLINE: 3516668]

**Tarrier 1998 *{published data only}***

Tarrier N, Yusupoff L, Kinney C, McCarthy E. Randomised controlled trial of intensive cognitive behaviour therapy for patients with chronic schizophrenia. *BMJ* 1998; **317**: 303–7. [MEDLINE: 9685273]

**Tashjian 2006 *{published data only}***

Tashjian RZ, Banerjee R, Bradley MP, Alford W, Fadale PD. Zolpidem reduces postoperative pain, fatigue, and narcotic consumption following knee arthroscopy: a prospective randomized placebo-controlled double-blinded study. *Journal of Knee Surgery* 2006; **19**: 105–11.

**Theroux 1993 *{published data only}***

Theroux MC, West DW, Corddry DH, Hyde PM, et al. Efficacy of intranasal midazolam in facilitating suturing of lacerations in preschool children in the emergency department. *Pediatrics* 1993; **91**(3): 624–7. [MEDLINE: 8441570]

**Thomas 1987**

Thomas KB. General practice consultations: is there any point in being positive?. *British Medical Journal* 1987;**294**: 1200–2. [MEDLINE: 3109581]

**Thomas 1999 *{published data only}***

Thomas VJ, Dixon AL, Milligan P. Cognitive-behaviour therapy for the management of sickle cell disease pain: an evaluation of a community based intervention. *British* *Journal of Health Psychology* 1999; **4**: 209–29.

**Tremeau 1992 *{published data only}***

Tremeau ML, Fontanie-Ravier P, Teurnier F, Demouzon J. Protocol of cervical maturation by acupuncture [Protocole de maturation cervicale par acupuncture]. *Journal de* *Gynecologie Obstetrique et Biologie de la Reproduction* 1992; **21**: 375–80. [MEDLINE: 1624722]

**Tsay 2003 *{published data only}***

Tsay SL, Chen ML. Acupressure and quality of sleep in patients with end-stage renal disease: a randomized controlled trial. *International Journal of Nursing Studies* 2003; **40**(1): 1–7.

**Tsay 2004 *{published data only}***

Tsay SL. Acupressure and fatigue in patients with end-stage renal disease-a randomized controlled trial. *International* *Journal of Nursing Studies* 2004; **41**: 99–106.

**Tuomilehto 1980 *{published data only}***

Tuomilehto J, Voutilainen E, Huttunen J, Vinni S, et al.Effect of guar gum on body weight and serum lipids in hypercholesterolemic females. *Acta Medica Scandinavica* 1980; **208**: 45–8. [MEDLINE: 7435246]

**Turner 1979 *{published data only}***

Turner RM, Ascher LM. Controlled comparison of progressive relaxation, stimulus control, and paradoxical intention therapies for insomnia. *Journal of Consulting* *and Clinical Psychology* 1979; **47**(3): 500–8. [MEDLINE:

393734]

**Vlaeyen 1996 *{published data only}***

Vlaeyen JWS, Teeken-Gruben NJG, Goossens MEJB, Rutten-van Mölken MPMH, et al.Cognitive-educational treatment of fibromyalgia: a randomized clinical trial. I. Clinical effects. *The Journal of Rheumatology* 1996; **23**(7): 1237–45. [MEDLINE: 8823699]

**Walton 1993 *{published data only}***

Walton RE, Chiappinelli J. Prophylactic penicillin: effect on posttreatment symptoms following root canal treatment of asymptomatic periapical pathosis. *Journal of Endodontics* 1993; **19**(9): 466–70. [MEDLINE: 8263455]

**Wang 1997 *{published data only}***

Wang B, Tang J, White PF, Naruse R, et al.Effect of the intensity of transcutaneous acupoint electrical stimulation on the postoperative analgesic requirement. *Anesthesia and* *Analgesia* 1997; **85**: 406–13.

**Werntoft 2001 *{published data only}***

Werntoft E, Dykes A. Effect of acupressure on nausea and vomiting during pregnancy. A randomized, placebocontrolled, pilot study. *The Journal of ReproductiveMedicine* 2001; **46**: 835–9.

**Whittaker 1963 *{published data only}***

Whittaker CB, Hoy RM. Withdrawal of perphenazine in chronic schizophrenia. *The British Journal of Psychiatry; the* *Journal of Mental Science* 1963; **109**:422–7.

**Wilcock 2008 *{published data only}***

Wilcock A,Walton A, Manderson C, Feathers L, El Khoury B, Lewis M, et al.Randomised, placebo-controlled trial of nebulised furosamide for breathlessness in patients with cancer. *Thorax* 2008; **63**: 872–5. [DOI: 10.1136/ thx.2007.091538]

**Williams 1988 *{published data only}***

Williams JM, Hall DW. Use of single session hypnosis for smoking cessation. *Addictive Behaviors* 1988; **13**:205–8. [MEDLINE: 3369332]

**Witt 2005 *{published data only}***

Witt C, Brinkhaus B, Jena S, Linde K, Streng A, Wagenpfeil S, et al. Acupuncture in patients with osteoarthritis of the knee: a randomised trial. *Lancet* 2005; **366**:136–43.

**Yates 1988 *{published data only}***

Yates RG, Lamping DL, Abram NL, Wright C. Effects of chiropractic treatment on blood pressure and anxiety: a randomized, controlled trial. *Journal of Manipulative and* *Physiological Therapeutics* 1988; **11**(6): 484–8. [MEDLINE:3075649]
