# Supplementary material for: Are Treatments More Effective than Placebos? A Systematic Review and Meta-Analysis
Source: PLoS One. 2013 May 15;8(5):e62599. doi: 10.1371/journal.pone.0062599 (PMC3655171; doi:10.1371/journal.pone.0062599)
Supplement: Flowchart S1 — PRISMA flowchart. (DOC) [file pone.0062599.s004.doc]

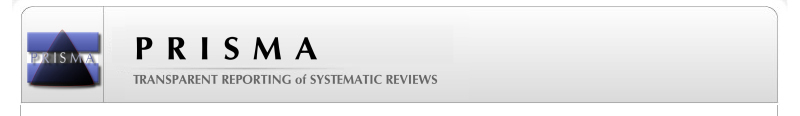
**PRISMA 2009 Flow Diagram**

**Screening**

**Included**

**Eligibility**

**Identification**

Records identified through database searching
(n = 202)

Additional records identified through other sources
(n = 0)

Records after duplicates removed
(n = 0)

Records screened
(n = 202)

Records excluded
(n = 0)

Full-text articles assessed for eligibility
(n = 202)

Full-text articles excluded, with reasons
(n = 50), data unavailable

Studies included in qualitative synthesis
(n = 152)

Studies included in quantitative synthesis (meta-analysis)
(n = 152)
